# Long-term impacts of salinity and temperature changes on *Brachionus calyciflorus* populations: understanding the role of intraspecific variability

**DOI:** 10.1007/s11356-025-35995-3

**Published:** 2025-01-29

**Authors:** Lishani Wijewardene, Cátia Venâncio, Rui Ribeiro, Isabel Lopes

**Affiliations:** 1 Faculty of Fisheries and Marine Sciences & Technology, Department of Limnology and Water Technology, University of Ruhuna, Matara, 81000 Sri Lanka; 2 CESAM & Department of Biology, University of Aveiro, 3810-193 Aveiro, Portugal; 3 Centre for Functional Ecology, Department of Life Sciences, University of Coimbra, 3000-456 Coimbra, Portugal

**Keywords:** Seawater intrusion, Climate change, Loss of genetic variability, Long-term exposure, Rotifera

## Abstract

Worldwide, many coastal freshwater ecosystems suffer from seawater intrusion. In addition to this stressor, it is likely that the biota inhabiting these ecosystems will also need to deal with climate change-related temperature fluctuations. The resilience of populations to long-term exposure to these stressors will depend on their genetic diversity, a key for their adaptation to changing environments. Accordingly, this study aimed to understand the long-term effects of salinity and temperature on the population density dynamics of the rotifer *Brachionus calyciflorus* by considering intra-specific variability. Six clonal lineages of *B. calyciflorus*, exhibiting differential lethal sensitivity (LC_50,24 h_) to salinity, were exposed for at least 34 days, to a control and to artificial seawater (at a conductivity corresponding to the LC_70,24 h_ for the most tolerant clonal lineage = 9.89 mS/cm), under three temperatures: 17, 20 (standard) and 23 °C. Long-term exposure to artificial seawater affected population densities, leading to the extirpation of some salinity-tolerant clonal lineages earlier than that of salinity-sensitive lineages. This inversion in short- and long-term sensitivity may suggest a higher susceptibility of populations when exposed to long periods of increased salinity. The negative effects caused by artificial seawater were enhanced at 17 °C and 23 °C, with an even earlier occurrence of extirpation of some clonal lineages, namely, two clonal lineages considered tolerant to artificial seawater. The results suggest the potential synergistic effects of the two abiotic stressors when combined. Overall, a lack of association between the clonal lineages’ short- and long-term sensitivity to salinity or their sensitivity to salinity under different temperature scenarios was observed. These results suggest an increased risk to the resilience of *B. calyciflorus* populations exposed to climate change-related scenarios of increased salinity and temperature fluctuations owing to an enhanced reduction in their genetic variability.

**Supplementary Information:**

The online version contains supplementary material available at 10.1007/s11356-025-35995-3.

## Introduction

Coastal freshwater ecosystems are vulnerable to environmental disruptions caused by climate change. Sea level rise and warming processes (often correlated events) are two of the main challenges imposed on the biota inhabiting these ecosystems (IPCC 2021). Recurrent episodes of salinization are expected in coastal freshwater ecosystems through storm-driven overtopping of seawater (leading to temporary salinization events), especially during the winter months, and groundwater intrusion (leading to temporary salinization events), especially during prolonged summer droughts. (IPCC 2021). In addition, IPCC's current scenarios of temperature increase by the end of the century have provided fragments of evidence, with medium confidence (according to the level of confidence expressed in IPCC 2021), that they could rise by 3 °C or more (IPCC 2021). Considering the ongoing climatic changes, the likelihood of co-occurrence of these two factors is high; therefore, their combined ecological impact to freshwater biota must be assessed. Studies that relate salinity and temperature stress factors are limited, and this number decreases when considering population genetic variability (Jeppesen et al. 2020; Cunillera-Montcusí et al. 2022). Population genetic diversity is nevertheless crucial for ecosystem structure and function, as it bolsters the adaptive capacity of populations (Convention on Biological Diversity 2010). Exposure to environmental changes may impact the genetic pool of freshwater species (Frankham 2005; Hoban et al. 2021; Loria et al. 2022), leading to genetic erosion and increased susceptibility to future stressors (e.g., Armbruster and Reed 2005 and references therein; Nowak et al. 2009; Ribeiro and Lopes 2013; Švara et al. 2021). Despite its relevance, genetic diversity is often neglected when conducting ecological risk assessments of contaminants or when planning biodiversity protection legislation and frameworks (Breitholtz et al. 2006; Hoban et al. 2021). Recent studies have shown that integrating genetic variability to study the effects of environmental disturbances, such as salinity and temperature, may more accurately estimate the ecological risks of factor interactions for freshwater biota. For example, Venâncio et al. (2023) studied laboratory populations of *Daphnia longispina* (with short-term differential lethal sensitivity to salinity) and found that long-term exposure to seawater caused a faster extirpation of salinity-tolerant clonal lineages than of salinity-sensitive ones, indicating no association between short-term and long-term sensitivity to this stressor. Furthermore, authors investigated interactive effects and suggested that salinity and temperature acted synergistically, increasing the negative effects of salinity (loss of most clonal lineages) under different temperature regimes (i.e., at 17 and 23 °C). The results of this work constitute a major advance in understanding how interactions between salinity and temperature can reduce the probability of survival of a population when considering intraspecific variability. Recognizing whether these patterns of effects exist in other taxa belonging to the same functional group of daphnia (e.g., rotifers) may allow us to understand how the genetic variability of other primary consumer communities is modulated under increased salinity and thermal stress and how this can impact future resilience. Furthermore, this knowledge is of much relevance when estimating impacts of environmental stressors at the community level. It is acknowledged that the existence of functional redundancy in natural communities supports their ecological resilience and stability (Fonseca and Ganade 2001; Biggs et al. 2020). Though, if species with equivalent functions in a community exhibit similar sensitivities and patterns of response to the same environmental stressors, then redundancy may decrease significantly in the community impairing its ecological resistance.

Rotifera, together with Cladocera, are among the main groups that dominate zooplankton communities, with important roles as food web regulators, exhibiting similar functions (ecological redundancy) in natural communities (being filter feeders and primary consumers) (Castro et al. 2005; Phan et al. 2021; Thackeray and Beisner 2024). Therefore, a comparative assessment of their responses to environmental stressors is of much relevance, as if they are similar, a significant component of the ecological redundancy may be lost in zooplankton communities, compromising their persistence and subsequently the ecological equilibrium at the ecosystem level. Though, rotifers have unique characteristics related to habitat preferences (niche partitioning-based, among others, on temperature) and life history strategies, such as long-term reproductive strategies, which can be influenced by exposure to salinity (Sarma et al. 2006). These are coupled with temperature (Huey and Kingsolver 2019) and may induce distinct outcomes from those exhibited by other ectotherms in both the short and long term (e.g., daphnids; Venâncio et al. 2023). This study aimed to confirm the hypothesis that short-term survival mechanisms are distinct from long-term resilience mechanisms in rotifers facing environmental disturbances (i.e., there is no correlation between short-term tolerance and long-term resilience; Lopes et al. 2005). If this hypothesis is true, the long-term resilience mechanisms of rotifers exposed to salinity and temperature are constantly trimmed by natural selection and have low heritability, compromising population viability in stressed coastal freshwater ecosystems. Considering that an adequate extrapolation of the potential effects of the combination of increased salinity and temperature at the ecosystem level is extremely reductive if based on an exercise carried out for a single group of organisms (Venâncio et al. 2023), the present study aimed to understand the effect of freshwater salinization at different temperature levels on the population density dynamics of the rotifer *Brachionus calyciflorus*, considering intraspecific variability. To study this main objective, the following two hypotheses were established: i) prolonged exposure to increased salinity and temperature would lead to a faster extinction of local rotifer populations (simulated in the laboratory by testing different clonal lineages of the rotifer) compared to prolonged exposure to increased salinity (controlled temperature); and ii) in a genetically diverse population of rotifers, the most salinity-sensitive clonal lineages would be the first to disappear regardless of the temperature level, and therefore, the persistence of the most salinity-tolerant clonal lineages would sustain the resilience of populations in salinity-impacted locations as well as temperature changes. With the knowledge generated, we intended to infer the possible consequences of the probability of extinction of *B. calyciflorus* clonal lineages on the resilience of populations. By considering the intraspecific variability in the response to these two stressors, this knowledge can contribute to the development of more precise and comprehensive protective measures in future climate change-induced salinization and warming scenarios. Therefore, this study aims to grow knowledge on the study of Venâncio et al. (2023) regarding the resilience of primary consumer communities to freshwater salinization and temperature changes by characterizing the response of an ecologically redundant species, thereby enhancing knowledge about the response of primary consumer taxa to the studied stressors, enabling more accurate extrapolations for the community and ecosystem levels.

## Materials and methods

### Artificial seawater

An artificial seawater stock solution was prepared by dissolving 33 g of artificial sea salt (Ocean Fish, Prodac International, Cittadella, Italy) in 1 L of ultrapure water (Milli-Q; Millipore, Burlington, MA, USA). The stock solution was then diluted with *B. calyciflorus* culture medium to obtain the salinity levels to be tested in toxicity assays (also simulating the occurrence of seawater dilution as it enters freshwater ecosystems). Artificial seawater was selected to perform this study over natural seawater to avoid the risk of using natural seawater contaminated with other pollutants, which would impair discriminating the toxicity induced by increased salinity or by unknown pollutants.

### Cultures of *Brachionus calyciflorus*

*Brachionus calyciflorus calyciflorus* Pallas, 1776 was selected as the model species of rotifers because it exhibits a parthenogenic reproductive strategy (allowing the maintenance, in the laboratory, of the exact same genotype over several generations) and is highly sensitive to salinity increases (Venâncio et al. 2019).

To establish cultures of different clonal lineages of *B. calyciflorus*, commercial cysts were acquired from RotoxKit F™ (MicroBio Tests Inc., Ghent, Belgium) and hatched following the standard procedure in RotoxKit F™ (MicroBioTests Inc., Ghent, Belgium). In brief, cysts were allowed to hatch at 23 °C for 24-h, at a constant light intensity of 3000–4000 lx in ASTM moderately hard synthetic freshwater medium (OECD 2004). Since only one organism hatches from each cyst, and cysts are the result of sexual reproduction, individuals from different cysts correspond to different genotypes, each one originating a clonal lineage. Sixteen clonal lineages were started from 16 cysts; each one was started with a single individual cyst, with less than 24-h old, that was then allowed to reproduce parthenogenetically (ensuring the maintenance of the clonal lineage for several generations). Each clonal lineage was cultured separately (at a maximum number of ten females/8 mL of medium) under controlled standard conditions of temperature (20 ± 1 °C) and photoperiod (16:8 h L:D) in reconstituted freshwater (ASTM medium), which was prepared according to the Rotoxkit F™ (MicroBioTests, Ghent, Belgium) procedure, and fed with the green microalgae *Raphidocelis subcapitata* at a concentration of 0.5 × 10^5^ cells/mL every day. The culture medium was renewed every other day. Each clonal lineage culture was checked daily to monitor for brood release, and for culture renewal, only neonates born from the 3rd or 4th asexual broods were used in the assays.

### Selection of clonal lineages of *B. calyciflorus* with extreme sensitivities to artificial seawater

Since the sensitivity of the 16 rotifer clonal lineages to artificial seawater was unknown, firstly, there was the need to characterize their short-term lethal sensitivity to artificial seawater. For this, standard acute 24-h assays were performed by following the Acute RotoxKit F™ protocol (MicroBio Tests, 1998). From the cultures of the 16 clonal lineages, neonates from 3rd or 4th broods, less than 24-h old, were isolated and exposed to a range of concentrations of artificial seawater, obtained by dilution with ASTM medium of the stock solution (33 g/L of artificial sea salt). Conductivity values will, from here onwards, be used as a surrogate measure of the concentrations of salinity to make comparisons with the published literature. The tested salinity levels were as follows: control (ASTM moderate hard water culture medium with a conductivity of ≈ 0.49 mS/cm), 3.50, 4.90, 6.86 and 9.60 mS/cm (Acute RotoxKit F™, MicroBioTests, Ghent, Belgium). Exposure was performed in 6-well plates, and each well was filled with 8 mL of the test solution. Five neonates from each clonal lineage were exposed per replicate, and three replicates were assembled per treatment and control. The assay was conducted at 20 ± 1 °C of temperature and photoperiod of 16:8 h L:D, with no medium renewal or food, as recommended by the Acute RotoxKit F™ protocol. At the end of the 24-h period, the number of immobile organisms (not exhibiting any movement for 15 s after gentle prodding) was recorded for each replicate. In Table S1 are described the LC_50_ and LC_70_ values obtained for all the clonal lineages of *B. calyciflorus*. From these, only six clonal lineages (representing the three lower and upper extremes of lethal sensitivity to artificial seawater, G, P, D, H, N, and F) were selected according to their estimated LC_50_ to increased levels of artificial seawater, to proceed with the study and perform the long-term assays described in the next section.

### Long-term assays with six clonal lineages of *B. calyciflorus*

Six clonal lineages of *B. calyciflorus*, differing in their short-term sensitivity to lethal levels of artificial seawater (D, G, P – sensitive and N, F, H – tolerant; Table S1), were selected to conduct the long-term assays. To conduct the long-term assays, each clonal lineage was exposed to a specific artificial seawater conductivity across three temperatures: (i) 17 ± 1 °C, (ii) 20 ± 1 °C, and (iii) 23 ± 1 °C. These temperatures were chosen based on the Sixth IPCC Assessment Report (2021) projections that foresee a global warming of 3.2 °C by the end of the century.

To initiate the assay, for each temperature and each clonal lineage of *B. calyciflorus*, five neonates less than 24-h old, from the 3rd to 5th broods, were assigned per replicate, which were filled with ASTM moderate hardwater medium. Assays were carried out in 6-well sterilized plastic plates, with each well corresponding to a replicate containing 8 mL of ASTM moderate hard water medium (with a conductivity of ≈ 0.48 mS/cm). Six replicates, in ASTM moderate hard water medium alone, were initially set for each temperature and clonal lineage of *B. calyciflorus*, and plates were maintained under a 16:8 h L:D photoperiod and to the temperature to which they were assigned as previously established. Organisms were fed daily with *R. subcapitata* (0.5 × 10^5^ cells/mL) until each population reached their carrying capacity (13 d for rotifers exposed at 17 °C, 10 d for rotifers exposed at 20 °C, and 8 d for rotifers exposed at 23 °C) (please see Figure S1). The daily counting of the total density of organisms in the plates allowed the determination of the carrying capacity, which was reached when, for a period of 3–4 days, the density counts varied within similar values. Populations that had already reached their carrying capacity were used to initiate the exposure experiments to ensure that there was no influence of other stressors, such as food limitation or competition (please see Figure S2).

After achieving the carrying capacity (being exposed in ASTM medium alone), populations of each replicate (each clonal lineage at each temperature) were assigned to each treatment: negative control (ASTM moderate hard water medium) and salt exposure which was the conductivity of artificial seawater that caused 70% mortality in the most tolerant clonal lineage (F) (LC_70_,_24 h_: 9.89 mS/cm; corresponding to 19% of seawater conductivity of ≈52 mS/cm). All controls and conductivity treatments were performed in triplicates. The photoperiod, feeding (provided to the organisms at each 24 h), and renewing practices were maintained as in laboratory cultures throughout the duration of the assay. The density of organisms was checked every 24 h until half of the clonal lineages (i.e., three clonal lineages) exposed to the artificial seawater treatments totally died (for all three temperatures, this time corresponded to 34 d), time at which the assay was considered finished. The exposure time causing 50% and 90% of mortality (LT_50_ and LT_90_), in each clonal lineage, was computed at 96-h of exposure (already considered as chronic exposure for rotifers; Preston et al. 2000) and at the end of the assay (corresponding to 624 h at 17 °C, 816 h at 20 °C, and 744 h at 23 °C; please see Results section for further details).

Conductivity, pH, and dissolved oxygen measurements were taken using scientific and technical equipment from Wissenschaftlich Technische Werkstätten (WTW, Weilheim, Germany), including the F330 conductivity meter, 330 pH meter, and OX330 oxygen meter. These measurements were recorded for *B. calyciflorus* in each treatment and for both new and old growth media during the renewal process.

### Data analysis

Short-term lethal conductivities of artificial seawater causing 50% and 70% of mortality (LC_50_ and LC_70_, respectively) were computed for each of the 16 clonal lineages of *B. calyciflorus* using the PriProbit software (Sakuma 1998).

For the selected six clonal lineages, the lethal time causing 50 and 90% mortality (LT_50_ and LT_90_, respectively), after 96-h of exposure and after the full duration of each long-term assay, were computed with exponential, logistic and Gompertz models using STATISTICA 7.0 (StatSoft, Hamburg, Germany), being chosen the model leading to the smallest relative spread:

$$Relative\;spread=\frac{Upper\;95{\%\;}confidence\;limit-Lower\;95\%\;confidence\;limit}{{LT}_{50} or {LT}_{90}}$$

Spearman’s correlations were computed to identify if significant associations existed between the LC_50_ or LC_70_ and the LT_50_, computed for the six clonal lineages of the rotifer i.e., to verify if clonal lineages with higher short-term lethal tolerance to artificial seawater, would be also the ones dying later in the long-term assay. For LT_90,_ such a correlation was not made, as these values are close to population extirpation.

To identify among-clonal differences in controls, population density at the end of each assay was checked to evaluate possible tolerance-associated fitness costs under no contamination stress using one-way analysis of variance (ANOVA). The survival time of the three most sensitive clones, under artificial seawater exposure, was compared using a two-way ANOVA. The LT_50_ values computed with data retrieved from the long-term assay, at 96-h and full assay duration times, were compared to identify any recovery ability along exposure time using one-way ANOVA.

The values of LT_90_ are the best estimation to understand the probability of extinction occurrence among lineages, where almost 90% of the individuals in the population died off and, thus, had the minimum possibility of recovering back. To test the effect of temperature on extinction risk within species under saline conditions, a one-way ANOVA was performed with the LT_90_ values obtained for each set of six clonal lineages under different temperature regimes. All data sets were tested for normality using the Kolmogorov–Smirnov test and homoscedasticity with the Bartlett test. Tukey’s and Dunnett´s multi-comparison tests were used after ANOVA to identify within-factor, among-level differences.

## Results

### Correspondence between the LC_50_ or LC_70_ and the LT_50_

No correlation was found between the LC_50_ or LC_70_, retrieved from short-term assays, and the LT_50,_ computed with data from 96-h or full assay-duration times of the long-term assay_,_ and obtained for each of the six clonal lineages of *B. calyciflorus* (Fig. S1). The computed Spearman correlations’ values ranged between −0.37 and 0.54 (0.30 ≤ *p* ≤ 1.0).

### Population densities: under exposure to control conditions (no artificial seawater)

The final densities of the six clonal lineages exposed under control conditions (with no artificial seawater) were compared within different temperatures (17 °C, 20 °C, and 23 °C; Fig. S2). No significant differences were found in the densities of the six clonal lineages of *B. calyciflorus* at 17 °C and 20 °C (Fig. S2a, S2b; Tukey’s test, p ≥ 0.09). However, at 23 °C, clonal lineages P, D, and H presented a significantly higher density, thus forming a distinct group, whereas clonal lineages G and F showed the lowest densities (Fig. S2c; Tukey’s, p ≤ 0.031).

### Population densities: under exposure to artificial seawater

**Fig. 1:**
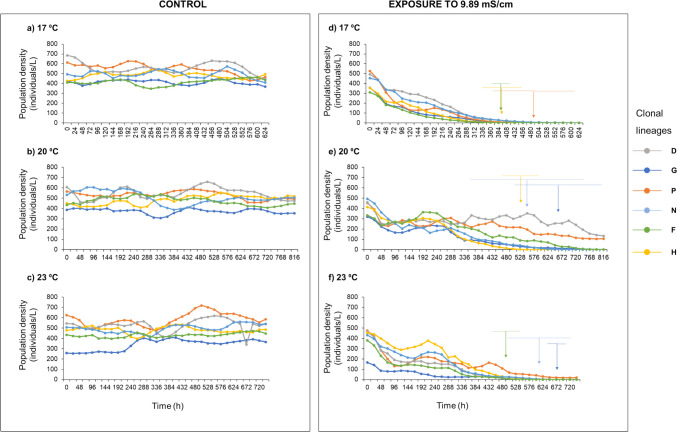
Population density (individuals/L) of six clonal lineages of *Brachionus calyciflorus*, presented as pooled running averages, exposed to control conditions (panel on the left) and exposed to artificial seawater (panel on the right) at three distinct temperatures (17 °C, 20 °C, and 23 °C). Exposure to artificial seawater corresponded to a conductivity level of 9.89 mS/cm. Three replicates were assembled per lineage and control or treatment. Vertical arrows indicate the average time of extinction (*n* = 3) for each one of the three clonal lineages to be extinct faster, the time at which the assay was ended. The horizontal lines aligned with the vertical arrows indicate the standard deviation (*n* = 3)

The density of the *B. calyciflorus* population in the absence of artificial seawater (i.e., in the controls) fluctuated slightly, during the assay (Fig. 1a, b, c). Exposure at a conductivity level of 9.89 mS/cm, resulted in different times in terms of the total duration of the assay (which was assumed to be complete when three clonal lineages were totally extinct): 624 h at 17 °C (Fig. 1d), 816 h at 20 °C (Fig. 1e), and 744 h at 23 °C (Fig. 1f). Regarding the time to extinction of the first three clonal lineages (time at which the assay was assumed to end), no significant differences were found between clonal lineages or temperatures (p > 0.05). However, some patterns of response were observed. For instance, clonal lineage H was the second and first clonal lineage to disappear at 17 °C and 20 °C (Fig. 1d, e), respectively, whereas clonal lineage F was the first to disappear at 17 °C and 23 °C (Fig. 1d, f). Both clonal lineages (H and F) were the most tolerant to artificial seawater based on the short-term LC_50_ values (Table S1). Clonal lineage P was the third to disappear at 17 °C and clonal lineage G was the third clonal lineage to disappear at 20 °C and 23 °C (Fig. 1d, e, f).

### Recovery ability across the long-term assay

**Fig. 2:**
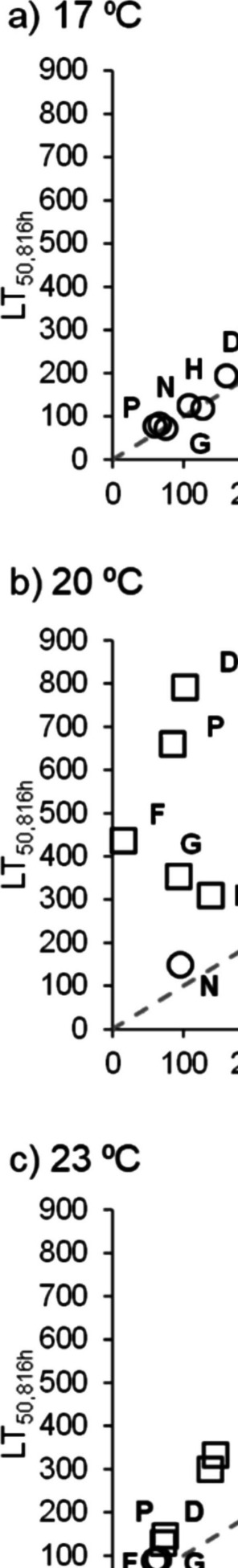
Relation between the mean lethal time (LT_50_, in hours) obtained during the long-term assay, after a 96-h exposure and at the end of the assay, for the six clonal lineages of the freshwater rotifer *Brachionus calyciflorus* exposed to artificial seawater (at a conductivity of 9.89 mS/cm), at three distinct temperatures (17 °C, 20 °C, and 23 °C; a to c, respectively). The grey dashed diagonal line indicates no recovery. Clonal lineages marked with circles are considered not different from the no recovery line; whilst those clonal lineages apart from the line, and marked with a square, are considered to disappear suddenly (if positioned on the right of the line, no clonal lineage showed this pattern) or to recover (if positioned on the left of the line)

The ability to recover was analyzed by plotting the LT_50_’s obtained from the long-term assay, after 96-h of exposure, against those obtained after the whole duration of the assay. The results are depicted in Fig. 2, and the details of the LT_50_ values are presented in Table S2.

*Brachionus calyciflorus* showed no recovery pattern in the long-term assay at 17 °C (Fig. 2a), whereas at 23 °C, clonal lineages D, H, and N showed a tendency to recover under long-term exposure, with the LT_50_ obtained at the end of the assay at 23 °C being 1.9-fold greater than the LT_50_ computed at 96-h of the long-term assay (Fig. 2c). At 20 °C, all clonal lineages of *B. calyciflorus* showed a tendency to recover under long-term exposure (apart from clonal lineage N), with the LT_50_ values at the end of the assay (816 h) being 1.5-fold greater than those LT_50_ computed after the 96-h period (Fig. 2b). The highest recovery was observed for clonal lineage F, which presented LT_50_ values computed at 96-h and at the end of the long-term assay, of 16.2 h and 432 h, respectively (Fig. 2b).

### Probability of extinction of clonal lineages under different temperature regimes

The LT_90_ is the best estimate to understand the probability of extinction among clonal lineages, since this value corresponds to almost 90% of the individuals in the population dying off, which matches the minimum possibility of recovering back.

In general, in *B. calyciflorus* clonal lineages, LT_90_ values increased with increasing temperature from 17 °C to 20 °C, with all LT_90_ values being statistically different (Tukey’s test, *p* < 0.05; Table 1). Though, in general, LT_90_ values decreased significantly from 20 °C to 23 °C for clonal lineages D, P, N, and F (Table 1). The LT_90_@17 °C ranged from 229 to 368 h, while the LT_90_@20 °C and LT_90_@23 °C ranged from 418 to 1037 h and 405 to 799 h, respectively. Overall, the clonal lineages of *B. calyciflorus* presented the highest within-population variability at 20 °C and 23 °C, with fold-changes in the LT_90_ of 2.5- and 2.0-fold, respectively (Table 1).

**Table 1:** Lethal time until the disappearance of 90% of the organisms (LT_90_, in hours) computed for six clonal lineages of *Brachionus calyciflorus* exposed to artificial seawater, under three distinct temperature regimes

	LT_90_ (h)		
Clonal Lineage	17 °C	20 °C	23 °C
D	**367.6**^a^ (337.8–397.3)	**885.9**^b^ (785.9–985.9)	**458.9**^a^ (390.8–526.9)
G	**282.0**^a^ (262.8–301.2)	**536.6**^b^ (464.8–608.4)	**472.6**^b^ (397.8–547.4)
P	**276.7**^a^ (241.2–312.2)	**1103.7**^c^ (797.2–1410.2)	**799.2**^b^ (526.3–1072.1)
N	**357.7**^a^ (336.5–378.9)	**555.7**^b^ (476.8–634.6)	**485.9**^a^ (419.4–552.4)
F	**229.0**^a^ (217.6–240.3)	**658.3**^c^ (592.8–723.7)	**405.1**^b^ (340.4–469.7)
H	**287.8**^a^ (262.1–313.5)	**418.5**^b^ (384.2–452.9)	**460.2**^b^ (423.3–497.0)

Values inside brackets represent the lower and upper confidence limits at 95%. Superscript letters (a, b, c) represent statistical differences between the LT_90_ (obtained from three replicates) within the respective clonal lineage between the three temperature regimes (Tukey’s post hoc, p < 0.05)

**Fig. 3:**
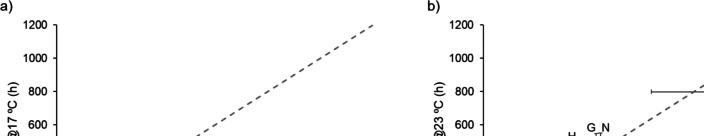
Lethal times until the disappearance of 90% of the population (LT_90_ in hours) computed for six clonal lineages of *Brachionus calyciflorus* after exposure to seawater treatment (9.89 mS/cm) and at three distinct temperatures. The dashed diagonal line indicates no difference in the LT_90_ between temperatures. If positioned at the left of the line, the LT_90_@17 °C/LT_90_@23 °C is higher than the LT_90_@20 °C; if positioned on the left of the line, the LT_90_@20 °C is higher than the LT_90_@17 °C/LT_90_@23 °C. Error bars indicate the confidence limits at 95%. The LT_90_@20 °C is the control temperature

Analyzing Fig. 3a, it is possible to confirm that all LT_90_@20 °C are higher than LT_90_@17 °C because all data points, and the respective 95% confidence limits, are positioned on the right of the diagonal line and with no overlapping. When plotting LT_90_@20 °C against LT_90_@23 °C, a greater diversity of responses was observed (Fig. 3b). Clonal lineages were positioned on the left of the diagonal line, but only the LT_90_@23 °C of clonal lineage H was significantly different from the LT_90_@20 °C, while the remaining lineages were positioned on the right of the diagonal line, with the LT_90_@20 °C of clonal lineages F and D being significantly higher than that computed at 23 °C (Fig. 3b).

## Discussion

Salinity tolerance and its effects on zooplankton physiology and life-history traits are diverse. Regarding the six clonal lineages of *B. calyciflorus* studied here, the LC_50,24 h_ values for artificial seawater ranged between 6.04 and 9.41 mS/cm. Venâncio et al. (2019) reported LC_50,24 h_ values of seawater and NaCl of 5.09 and 4.01 mS/cm, respectively, for *B. calyciflorus* obtained from hatched cysts in the laboratory. Other LC_50,24 h_ recorded for osmotic stress induced by NaCl in this species indicated values of 3.75 g/L (approximately 7.42 mS/cm) (Peredo-Álvarez et al. 2003), and up to 5 g/L (9.9 mS/cm) in laboratory cultures of *B. calyciflorus* initiated with individuals of hatched resting eggs, as done in the present work (Greenwald and Hurlbert 1993; Snell et al. 1991). Thus, the LC_50,24 h_ values of this study lay in the same range as those reported in previous studies. Zooplanktonic species are considered poor osmoregulators. Thus, previous studies have pointed to the failure of homeostatic balance as well as the energy dispended with that process as the most likely causes of low reproductive rates, high juvenile mortality, and later adult mortality in salinity-stressed freshwater zooplankton (Peredo-Álvarez et al. 2003; Smirnov 2017). Comparing the sensitivity of the tested clonal lineages of *B. calyciflorus* with other planktonic primary consumer species, namely cladocerans, it is perceived that they seem to have a higher lethal tolerance to increased salinity (caused by seawater). For example, the highest LC_50,24 h_ registered for *D. longispina* (7.04 mS/cm) is slightly smaller than that registered in the present work for *B. calyciflorus* (9.41 mS/cm), which may just be due to the small sampling size of clonal lineages in both species (*n* = 6) (Venâncio et al. 2018). The slight differential sensitivity between these two primary consumers taxa may also be related to the structures involved in ionic regulation. In rotifers, ionic regulation is assured by a rudimentary protonephridia system, whereas in daphnids, it is mediated by epipodites and changes throughout ontogenetic development, which seems to require more investment on the part of daphnids (Aladin 1991; Aladin and Potts 1995; Thorp and Covich 2009).

Furthermore, it is evident that temperature is a key factor for poikilothermic species, increases in the temperature of the environment has a direct effect on their life history and population increment, as stated previously by other authors (e.g., Anderson-Carnahan 1994; El-Gamal et al. 2014; Anitha et al. 2016; Adamczuk 2020). For instance, from 17 °C or 20 °C to 23 °C, some *B. calyciflorus* clonal lineages (like P) were able to reach a higher population density in a shorter time period. This may be related to the occurrence of a decrease in mean generation time and an increase in other life history traits (such as the number of neonates produced per day, net reproductive rate, and innate capacity of population increase rate) associated with increased temperature. Kauler and Enesco (2011) found a positive association between these life-history parameters and increased temperatures in *B. calyciflorus*. Half of the *B. calyciflorus* populations (namely of P, D, and H clonal lineages) increased in population density when exposed to 23 °C without the presence of artificial seawater. These results could be explained by the fact that rotifers exhibit high phenotypic plasticity with respect to temperature tolerance (Paraskevopoulou et al. 2019). For example, *B. calyciflorus* is able to tolerate temperatures within the range of 15–31 °C (Arimoro 2006), and their optimal temperatures have been reported differently in the literature, such as 25 °C (Arimoro 2006) and 29 °C (Anitha et al. 2016).

Understanding the effects of the joint exposure to multiple stressors, in this case salinity and temperature, on zooplankton is important under current environmental conditions, where such multiple stressors scenarios are evident and increase with continuous human intervention in the environment. When considering the exposure of rotifers to artificial seawater (9.89 mS/cm) under different temperature levels, the time taken for the first three clonal lineages to disappear was the lowest at the lowest temperature (624 h at 17 °C), followed by the highest temperature (744 h at 23 °C), and the intermediate temperature regime registered the highest time to have three clonal lineages totally extinct (816 h at 20 °C), despite no significant differences were found among clonal lineages nor temperature regimes. These results suggest a higher tolerance of the rotifers to salinity at the intermediate tested temperature (20 ºC). The pattern of these results agrees with studies that assessed the influence of temperature in the sensitivity of this species of rotifer to other chemicals. Snell et al. (1991) assessed the influence of temperature in the tolerance of *B. calyciflorus* to copper and sodium pentachlorophenate, and reported that the lowest values of LC_50_s for these two chemicals were estimated for rotifers exposed at the lowest (10 ºC) and highest (35 ºC) tested temperatures comparatively to those exposed at intermediate temperature values (15 to 25 ºC). However, it must be noted that in the literature examples of different patterns on the influence of temperature in the toxicity of chemicals to rotifers have been reported, showing a dependency on the species and compound being tested (e.g., Velasco et al. 2019; and references therein). In the present work, it is hypothesised that the lowest tolerance of rotifers at 17 ºC may be associated with the lowest ability of rotifers to thrive under lower thermal regimes (Paraskevopoulou et al. 2020). Low water temperatures (e.g., 16 °C) have shown to be associated with reduced fecundity, lower growth rates, longer lifespans, elevated lipid and protein synthesis and metabolism for energy balance in populations of rotifers (Kauler and Enesco 2011; Fielder et al. 2000; Lee et al. 2020). These alterations in the life cycle and physiology of the rotifers could have limited their response and recovery from the exposure to increased salinity levels, namely, reduced fecundity and population growth rate could have contributed to impairing the capacity to restore the number individuals in the population at a rate that compensated for their elimination due to mortality induced by increased salinity. On the other hand, it is known from the scientific literature that different physiological mechanisms are involved in the response to salinity and high temperature, in the former being activated osmoregulatory responses and in the later heat shock proteins (e.g., Thabet et al. 2017; Paraskevopoulou et al. 2020), which may interact additively, thus explain the lower tolerance of rotifers to salinity under the exposure scenario of 23 ºC comparatively to that at 20 ºC.

Longer juvenile periods and prolongation of the mean lifespan at 20 °C have been reported in previous studies, which explains the longer time until the disappearance of the first three clonal lineages of rotifers at 20 °C (Trinh and Duong 2023). A similar trend was exhibited in the results obtained for all clonal lineages exposed to 20 °C, which suggests that salinity-stressed *B. calyciflorus* populations are less likely to lose clonal lineages (and, thus, have their genetic pool eroded) than salinity-stressed populations exposed to the other two temperature levels (17 °C and 23 °C), and the rotifer populations obtained at 17 °C and 23 °C, 17 °C-exposed rotifer populations are expected to exhibit the highest probability to have reduced genetic diversity in a shorter time due to the loss of clonal lineages.

A lack of correspondence between short-term lethal and long-term salt tolerance and between salt tolerance and extirpation of the clonal lineages was notable in the present study. For example, the first extinct *B. calyciflorus* clonal lineages were those presenting intermediate to high short-term lethal tolerance to salinity (H and F, based on their LC_50_) at 17 °C. This lack of association between short-term lethal tolerance and long-term sub-lethal tolerance to chemical stress has been reported in other studies (Barata et al. 2000; Lopes et al. 2005). For example, Lopes et al. (2005) reported no association between lethal and sublethal sensitivity to chemicals among clonal lineages of the cladoceran *D. longispina*, suggesting that this result was related to different genes regulating the responses at those two levels. At short-term lethal responses, few genes are most probably related to more specific mechanisms (e.g. changes in enzymatic activities) are involved, while at sublethal effects many genes related with more general responses (feeding, reproduction) control the responses (Hoffmann and Parsons 1997; Barata et al. 2000; Lopes et al. 2005). Furthermore, in the study performed by Venâncio et al. (2023) with clonal lineages of *D. longispina*, similar results to the ones obtained in the present study with rotifers were obtained: intermediate and tolerant lineages of *D. longispina*, E99 and N35, were the first lineages to disappear in the long-term assay at 17 °C. This reinforces the hypothesis stating that short-term lethal responses are controlled by genes that are different from those involved in long-term responses to chemical stress. This lack of correspondence between higher lethal tolerance and higher lifespan or probability of survival upon long-term exposure to salinity at different temperature regimes may have harsher consequences on the genetic pool of the populations and their resilience than those assumed by single or even combined stressor(es) studies using a single clonal lineage (e.g., Loureiro et al. 2015; De Coninck et al. 2013; Chain et al. 2019).

Recovery is a key mechanism by which organisms thrive and adjust to changing environments. If a capacity for long-term recovery to increased salinity of the clonal lineages of *B. calyciflorus* exists, it would constitute a win–win situation because the resilience of the population would be ensured while maintaining its genetic pool (and therefore its capacity to respond to future scenarios of environmental variability). For *B. calyciflorus*, especially at 20 °C, a higher variability in long-term recovery abilities were observed, with tolerant or intermediate lineages detached from others (such as D and P). Short-term LT_50_ values for *B. calyciflorus* were very low compared with long-term LT_50_ values, indicating their ability to recover from long-term exposure, even when the population experienced deadly effects in the initial phase. This response pattern was not observed in the study of Venâncio et al. (2023), who reported no recovery ability of *D. longispina* clonal lineages chronically exposed to artificial seawater at different temperature levels (17 °C, 20 °C, and 23 °C) (i.e., LT_50_ computed on short-term exposures were very similar to those computed for long-term exposures). Aside results of Coldsnow et al. (2017) with *D. pulex*, other works on zooplankton species (*Simocephalus vetulus*) have demonstrated long-term evolved halotolerance within natural populations (Loureiro et al. 2013). These different responses between rotifers and daphnias might be explained by the “phenotypic plasticity” of the *B. calyciflorus* populations. The lack of information regarding differential clonal tolerance of *B. calyciflorus* to salinity alone or in combination with temperature hinders the comprehensive understanding of the results obtained, an observation also made by Ramos-Rodríguez et al. (2020). One may also support the data obtained from studies that reflect the wide phenotypic plasticity within *Brachionus* sp. populations. For instance, when subjected to food and temperature variations, it was confirmed that within the same population, rotifers may have substantially differences amongst them (different phenotypes and physiological changes) (Ramos-Rodríguez et al. 2020). Such characteristics are fundamental to coping with changing environments and are thus the cornerstone of population resilience.

Our study provides insights into zooplankton dynamics under multiple stressors, which occur abruptly and intensify with climate change. Within climate change scenarios, temperature increases may be rather gradual, unlike salinization events that might be sudden, for instance, if scenarios of seawater overtopping due to sudden extreme storm events. The simulation of extinction scenarios presented here by computation of the LT_90_ might be a good surrogate to assess how vulnerable a specific clonal lineage is to abiotic stressor combinations. However, research often neglects sudden changes or abrupt regime shifts, and thus, the probability of extinction. The probability of extinction in *B. calyciflorus* decreased significantly from 17 °C to 20 °C in all clonal lineages, whereas it increased (in four lineages) or was maintained (in two lineages) from 20 °C to 23 °C. But it must be highlighted that when comparing the estimated probability of extinction at 17 °C and 23 °C with that at 20 °C, it was lower in the latter temperature scenario. The IPCC report highlights the increasing frequency and magnitude of extreme events (e.g., floods, droughts, storm surges) and sea level rise, which may lead to the salinization of coastal freshwater ecosystems (IPCC 2021). The results obtained in the present study suggest that salinization events occurring in both winter (e.g., seawater overtopping by storm surges) and summer (e.g., droughts) temperatures may lead to accelerated genetic erosion in *B. calyciflorus* populations. In a study by Venâncio et al. (2023) on *D. longispina*, the probability of extinction increased in five out of the six tested clonal lineages as the temperature increased from 17 to 23 °C. Here again, the reflection of the higher phenotypic plasticity of *B. calyciflorus* might be brought to light and, moreover, that in *D. longispina,* loss of resilience under extreme salinization scenarios boosted by temperature is very likely (Venâncio et al. 2023).

*B. calyciflorus*, the model species used in the present study, is revealing impacts of multiple stressors on genetic variability of a population. *B. calyciflorus* has a parthenogenetic mode of reproduction, a short life span, which was advantageous to lab-culturing of the same exact genotype (clonal lineage). Therefore, the maternal and environmental effects were eliminated from this study. This leads to the assumption that differences observed within clonal lineages are genetically determined variations and that observed population density fluctuations are the response of the clonal lineage solely to selective pressure, that is, salinity. It is now well known that a direct relationship exists between individual tolerance limits and phenotypic plasticity and further connectivity of plasticity and adaptation to novel environments (Latta et al. 2012). Genetic structure and diversity are important factors in invertebrate populations that have limited dispersal capabilities (Scheffer et al. 2006). This study confirmed that temperature itself may, in the long term, be a selective pressure to rotifer populations, and in combination with increased salinity, might worsen and/or speed the loss rate of clonal lineages, and thus, the genetic diversity in natural populations. As genetic diversity is a crucial factor for a population’s resilience, its reduction may further affect the fitness, environmental plasticity, co-tolerance, and trade-off mechanisms of the population, ultimately leading to the extinction of species (Chen et al. 2012; Ribeiro & Lopes 2013; Venâncio et al. 2023). Furthermore, by integrating the results obtained for *B. calyciflorus* with those of Venâncio et al. (2023) for *D. longispina*, which also reported a faster extirpation of lethal salinity-tolerant clonal lineages compared to salinity-sensitive clonal lineages after long-term exposure, it was hypothesized that the two taxa would be at a higher risk of extinction in scenarios of combined salinity and temperature stress. Considering that the two species are key representatives of primary consumers, thus playing similar functions in freshwater ecosystems, it is foreseen that their higher risk of extinction will repercute a lower functional redundancy in these communities, which can impact the stability and resilience of freshwater ecosystems.

## Conclusion

Climate change-induced salinization events are evident in freshwater ecosystems and can be intensified by extreme events, such as summer droughts and winter sea flooding. The results of the long-term laboratory assays with clonal lineages of *B. calyciflorus* (selected because of their differential short-term lethal sensitivity to salinity), combining a salinization scenario with different temperature levels, indicated that scenarios of seawater intrusion (and subsequent increased salinity) due to summer droughts (23 °C) and winter sea flooding (17 °C) events can further enhance the loss of genetic variability (the cornerstone of population resilience) of the rotifer population, compared with the exposure temperatures of 20 °C. Moreover, clonal lineages presenting higher or intermediate tolerance to salinity, where the first ones to disappear; thus, it is anticipated that the loss of genetic variability within the population might be higher than expected and will increase substantially the susceptibility of the rotifer population to future stressors and/or environmental changes. These are groundbreaking results because results from ecotoxicological assays combining abiotic factors almost always rely on a single lineage; therefore, this work has a pivotal role in understanding and predicting a population's local extinction and/or resilience under climate change scenarios related to increased salinity and temperature.

## Supplementary Information

Below is the link to the electronic supplementary material.

Supplementary file1 (DOCX 230 KB)

## Data Availability

The datasets generated during and/or analysed during the current study are available from the corresponding author on reasonable request.
