# Supplementary material for: Long-term impacts of salinity and temperature changes on Brachionus calyciflorus populations: understanding the role of intraspecific variability
Source: Environ Sci Pollut Res Int. 2025 Jan 29;32(8):4489–501. doi: 10.1007/s11356-025-35995-3 (PMC11850573; doi:10.1007/s11356-025-35995-3)
Supplement: Supplementary file 1 — Supplementary file1 (DOCX 230 KB) [file 11356_2025_35995_MOESM1_ESM.docx]

**Supplementary information**

**Tables**

**Table S1** - Conductivities (mS/cm) causing x% of mortality, after an exposure of 24 hours (LC_x,24_h) to artificial seawater, for each of the sixteen clonal lineages of *Brachionus calyciflorus*. The 95% confidence limits (95% CL) are depicted inside brackets.

|  | Clonal lineage  of *Brachionus calyciflorus* | LC_50,24h_ mS/cm  (95% CL) | LC_70,24h_ mS/cm  (95% CL) |
| --- | --- | --- | --- |
|  | **Q** | 5.74  (-) | 7.23  (-) |
|  | **L** | 5.98  (5.31-7.07) | 7.65  (6.57-10.07) |
|  | **D** | 6.04  (-) | 7.60  (-) |
|  | **G** | 6.06  (5.26-7.11) | 7.64  (6.60-9.82) |
|  | **I** | 6.38  (-) | 7.74  (-) |
|  | **P** | 6.56  (5.95-7.30) | 7.47  (6.78-8.66) |
|  | **E** | 6.79  (-) | 8.16  (-) |
|  | **B** | 6.85  (-) | 8.33  (-) |
|  | **M** | 7.04  (6.34-7.97) | 8.16  (7.32-9.76) |
|  | **K** | 7.08  (-) | 8.66  (-) |
|  | **C** | 7.37  (-) | 8.31  (-) |
|  | **N** | 8.28  (7.55-9.25) | 9.24  (8.43-10.9) |
|  | **J** | 8.31  (-) | 10.27  (-) |
|  | **F** | 8.34  (7.39-10.0) | 9.89  (8.61-13.2) |
|  | **O** | 8.37  (-) | 11.46  (-) |
|  | **H** | 9.41  (9.13-9.68) | 9.66  (9.38-9.95) |

**Table S2** - Median lethal time (LT_50_, in hours) and respective 95% confidence limits, computed for the six clonal lineages of *Brachionus calyciflorus* under exposure to a conductivity level of 9.66 mS/cm of artificial seawater, under three temperatures. The short-term exposure refers to an exposure period of 96 h for all temperatures, and the long-term exposure to an exposure period of 624 h (at 17 °C), 816 h (at 20°C) and 744 (at 23 °C).

|  | **LT_50_ in hours** | | | | | | |
| --- | --- | --- | --- | --- | --- | --- | --- |
|  | | **17 °C** | | **20 °C** | | **23 °C** | |
| **Clonal lineages** | | **96-h** | **End of assay** | **96-h** | **End of assay** | **96-h** | **End of assay** |
| **D** | | 159  0 – 445 | 195  176 – 213 | 103  0 – 327 | 790  754 – 826 | 74.2  39.7 – 109 | 145  111 – 179 |
| **G** | | 67.6  17.8 - 118 | 83.2  76.0 – 90.5 | 94.1  61.9 – 126 | 353  319 – 387 | 70.2  0 – 151 | 128  97.2 – 160 |
| **P** | | 59.5  50.4 – 68.6 | 78.0  65.8 – 90.1 | 85.4  0 – 251 | 659  577 – 740 | 60.0  52.9 – 67.0 | 87.0  34.4 – 140 |
| **N** | | 106  49.6 – 163 | 125  114 – 136 | 96.3  80.4 – 112 | 149  111 – 186 | 138  36.1 – 241 | 299  269 – 329 |
| **F** | | 73.3  31.7 - 115 | 73.1  68.9 – 77.3 | 16.2  0 – 98.7 | 435  403 - 467 | 64.8  46.2 – 83.4 | 90.9  64.7 - 117 |
| **H** | | 127  0 – 380 | 119  104 – 134 | 139  0 – 282 | 309  292 – 326 | 146  129 – 163 | 332  314 – 351 |

**Figures**


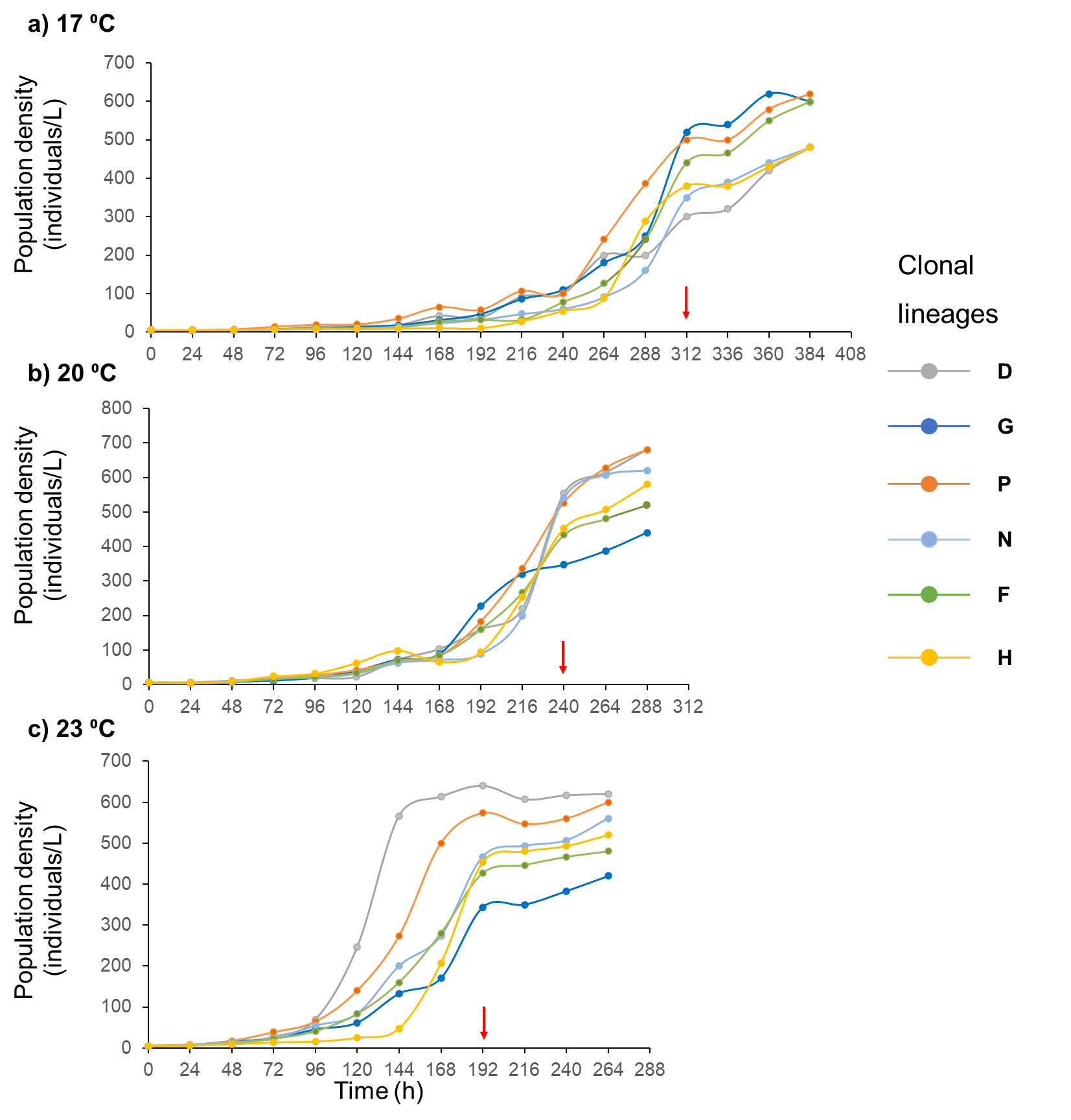


**Figure S1** - Population density (individuals/L) of six clonal lineages of *Brachionus calyciflorus*, presented as pooled running averages, from start of cultures until reach the carrying capacity under control conditions before exposed to artificial seawater at three distinct temperatures (17 °C, 20 °C, and 23 °C). Six replicates were assembled per lineage. Vertical red arrows indicate the average time taken to reach carring capacity (n=6) for each one of the clonal lineages. Each rotifer population reached their carrying capacity at 13 d for rotifers exposed at 17 °C, 10 d for rotifers exposed at 20 °C, and 8 d for rotifers exposed at 23 °C, respectively.


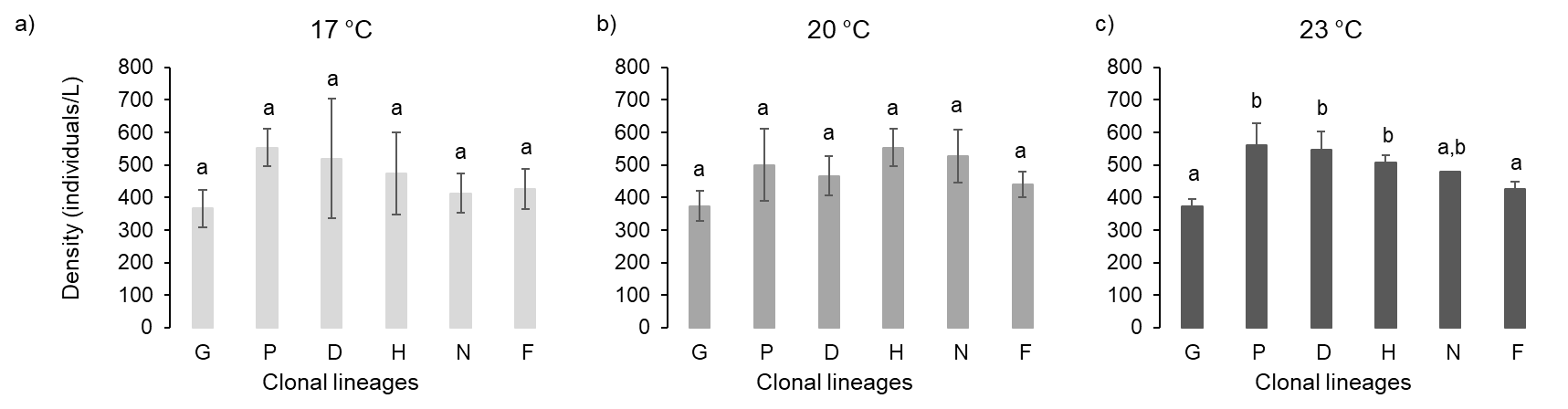


**Figure S2** - Average population densities for the six clonal lineages of *Brachionus calyciflorus,* under control conditions, at three distinct temperatures. Error bars correspond to the standard deviation. Letters (a, b) represent homogenous groups within temperatures (Tukey’s test, p < 0.05).
